# Congenital Diarrhea and Cholestatic Liver Disease: Phenotypic Spectrum Associated with MYO5B Mutations

**DOI:** 10.3390/jcm10030481

**Published:** 2021-01-28

**Authors:** Denise Aldrian, Georg F. Vogel, Teresa K. Frey, Hasret Ayyıldız Civan, Aysel Ünlüsoy Aksu, Yaron Avitzur, Esther Ramos Boluda, Murat Çakır, Arzu Meltem Demir, Caroline Deppisch, Hans-Christoph Duba, Gesche Düker, Patrick Gerner, Jozef Hertecant, Jarmila Hornová, Simone Kathemann, Jutta Koeglmeier, Arsinoi Koutroumpa, Roland Lanzersdorfer, Raffi Lev-Tzion, Rosa Lima, Sahar Mansour, Manfred Meissl, Jan Melek, Mohamad Miqdady, Jorge Hernan Montoya, Carsten Posovszky, Yelena Rachman, Tania Siahanidou, Merit Tabbers, Holm H. Uhlig, Sevim Ünal, Stefan Wirth, Frank M. Ruemmele, Michael W. Hess, Lukas A. Huber, Thomas Müller, Ekkehard Sturm, Andreas R. Janecke

**Affiliations:** 1 Department of Pediatrics I, Medical University of Innsbruck, A-6020 Innsbruck, Austria; denise.aldrian@i-med.ac.at (D.A.); georg.vogel@i-med.ac.at (G.F.V.); teresita-tea@web.de (T.K.F.); thomas.mueller@i-med.ac.at (T.M.); 2 Division of Cell Biology, Biocenter, Innsbruck Medical University, A-6020 Innsbruck, Austria; 3 Department of Pediatric Gastroenterology, Hepatology and Nutrition, Health Science University, Sadi Konuk Education and Research Hospital, 34147 Istanbul, Turkey; hasretayyildiz@yahoo.com; 4 University of Health Sciences, Sami Ulus Maternity and Child Health and Diseases Training and Research Hospital, 06120 Ankara, Turkey; ayselun@gmail.com; 5 Division of Gastroenterology, Hepatology and Nutrition, The Hospital for Sick Children, Toronto, ON M5G 0A4, Canada; yaron.avitzur@sickkids.ca; 6 Intestinal Rehabilitation Unit, Pediatric Gastroenterology and Nutrition Unit, University Hospital La Paz, 28046 Madrid, Spain; erboluda@salud.madrid.org; 7 Departments of Pediatric Gastroenterology Hepatology and Nutrition, Faculty of Medicine, Karadeniz Technical University, 61080 Trabzon, Turkey; muratcak@hotmail.com; 8 Ankara Child Health and Diseases, Training and Research Hospital, Pediatric Gastroenterology, 06130 Ankara, Turkey; arzuyusufoglu@yahoo.com; 9 Universitätsklinik für Kinder- und Jugendmedizin Tübingen, Pädiatrische Gastroenterologie und Hepatologie, Hoppe-Seyler-Straße 1, 72076 Tübingen, Germany; Caroline.Deppisch@med.uni-tuebingen.de; 10 Department of Medical Genetics, Kepler University Hospital, School of Medicine, Johannes Kepler University, A-4020 Linz, Austria; hans-christoph.duba@kepleruniklinikum.at; 11 Department for Pediatric Gastroenterology and Hepatology, University Children’s Hospital Bonn, 53127 Bonn, Germany; gesche.dueker@ukbonn.de; 12 Department of Pediatrics and Adolescent Medicine, Faculty of Medicine, Medical Center, University of Freiburg, 79106 Freiburg, Germany; patrick.gerner@uniklinik-freiburg.de; 13 Genetics/Metabolics Service, Tawam Hospital, Al Ain 15258, United Arab Emirates; jhertecant@seha.ae; 14 Department of Pediatrics, Faculty of Medicine, Comenius University, National Institute of Children Diseases, 814 99 Bratislava, Slovakia; jarmila.hornova@gmail.com; 15 Department for Pediatric Nephrology, Gastroenterology, Endocrinology and Transplant Medicine, Clinic for Pediatrics II, University Children’s Hospital Essen, University Duisburg-Essen, 45147 Essen, Germany; simone.kathemann@uk-essen.de; 16 Department of Paediatric Gastroenterology, Unit of Nutrition and Intestinal Failure Rehabilitation, Great Ormond Street Hospital for Sick Children NHS Foundation Trust, Great Ormond Street, London WC1N 3JH, UK; jutta.koeglmeier@gosh.nhs.uk; 17 Aghia Sofia Children’s Hospital, Neonatal Intensive Care Unit B, 115 27 Athens, Greece; koutroumpaarsinoi@gmail.com; 18 Department of Paediatrics and Adolescent Medicine, Johannes Kepler University Linz, A-4020 Linz, Austria; Roland.Lanzersdorfer@kepleruniklinikum.at; 19 Pediatric Gastroenterology, Shaare Zedek Medical Center, Jerusalem 9103102, Israel; raffilv@szmc.org.il (R.L.-T.); jryelanar@gmail.com (Y.R.); 20 Unidade de Gastrenterologia Pediátrica-Centro Hospitalar do Porto, 4099-001 Porto, Portugal; rosalima@netcabo.pt; 21 SW Thames Regional Genetics Service, St. George’s University NHS Foundation Trust, London SW17 0QT, UK; sahar.mansour@nhs.net; 22 Department of Neonatology, Johannes Kepler University Linz, A-4020 Linz, Austria; Manfred.Meissl@kepleruniklinikum.at; 23 Pediatric Gastroenterology, Department of Pediatrics, Faculty of Medicine in Hradec Králové, Charles University, 110 00 Prague, Czech Republic; jan.melek1@gmail.com; 24 Department of Pediatric, Sheikh Khalifa Medical City, College of Medicine & Health Sciences, Khalife University, Abu Dhabi 127788, United Arab Emirates; mmiqdady@seha.ae; 25 Hospital Universitario San Vicente de Paúl, Medellín, Antioquia 50022, Colombia; jorgeh.montoya@gmail.com; 26 Department of Pediatrics and Adolescent Medicine, University Medical Center Ulm, Eythstr. 24, 89075 Ulm, Germany; Carsten.Posovszky@gmx.de; 27 First Department of Pediatrics, Athens University Medical School, 11527 Athens, Greece; siahan@med.uoa.gr; 28 Emma Children’s Hospital/AMC, 1105 Amsterdam, The Netherlands; m.m.tabbers@amsterdamumc.nl; 29 Translational Gastroenterology Unit, University of Oxford, Oxford OX3 9DU, UK; holm.uhlig@ndm.ox.ac.uk; 30 Department of Pediatrics, University of Oxford, Oxford OX3 9DU, UK; 31 Ankara Child Health and Diseases, Training and Research Hospital, Neonatology, 06120 Ankara, Turkey; sevimunal@yahoo.com; 32 Department of Paediatrics, Helios Medical Centre Wuppertal, Witten-Herdecke University, 58455 Witten, Germany; stefan.wirth@helios-gesundheit.de; 33 Assistance Publique–Hôpitaux de Paris, Hôpital Universitaire Necker Enfants, Malades Service de Gastroentérologie, Hépatologie et Nutrition Pédiatrique, 149, Rue de Sèvres, 75015 Paris, France; frank.ruemmele@aphp.fr; 34 Paediatrics at the Medical Faculty, Université de Paris, 75005 Paris, France; 35 Institute of Histology and Embryology, Medical University of Innsbruck, A-6020 Innsbruck, Austria; michael.hess@i-med.ac.at; 36 Division of Cell Biology, Medical University of Innsbruck, A-6020 Innsbruck, Austria; lukas.a.huber@i-med.ac.at; 37 Austrian Drug Screening Institute, ADSI, A-6020 Innsbruck, Austria; 38 Children’s Hospital Tübingen, 72076 Tübingen, Germany; 39 Division of Human Genetics, Medical University of Innsbruck, A-6020 Innsbruck, Austria

**Keywords:** congenital diarrheal diseases, enteropathy, microvillus inclusion disease, MYO5B, myosin Vb, progressive familial intrahepatic cholestasis, PFIC, genotype–phenotype correlation, lack of protein, tail domain

## Abstract

Myosin Vb (MYO5B) is a motor protein that facilitates protein trafficking and recycling in polarized cells by RAB11- and RAB8-dependent mechanisms. Biallelic MYO5B mutations are identified in the majority of patients with microvillus inclusion disease (MVID). MVID is an intractable diarrhea of infantile onset with characteristic histopathologic findings that requires life-long parenteral nutrition or intestinal transplantation. A large number of such patients eventually develop cholestatic liver disease. Bi-allelic MYO5B mutations are also identified in a subset of patients with predominant early-onset cholestatic liver disease. We present here the compilation of 114 patients with disease-causing MYO5B genotypes, including 44 novel patients as well as 35 novel MYO5B mutations, and an analysis of MYO5B mutations with regard to functional consequences. Our data support the concept that (1) a complete lack of MYO5B protein or early MYO5B truncation causes predominant intestinal disease (MYO5B-MVID), (2) the expression of full-length mutant MYO5B proteins with residual function causes predominant cholestatic liver disease (MYO5B-PFIC), and (3) the expression of mutant MYO5B proteins without residual function causes both intestinal and hepatic disease (MYO5B-MIXED). Genotype-phenotype data are deposited in the existing open MYO5B database in order to improve disease diagnosis, prognosis, and genetic counseling.

## 1. Introduction

Microvillus inclusion disease (MVID) is a severe congenital enteropathy with intractable watery diarrhea, most often starting in the first days after birth but at times starting within the first months of life. Histopathology demonstrates hypoplastic villous atrophy; characteristic cytoplasmic inclusions of brush border microvilli and “secretory granules”, a subapical accumulation of aberrant vesicular compartments, are detected by electron microscopy [1]. Both immunohistochemistry and electron microscopy reveal the mislocalization of brush border transporter proteins and rarefication of microvilli ultimately leading to osmotic and secretory diarrhea, malabsorption, and failure to thrive. The prognosis is generally poor and continuous total parenteral nutrition (TPN) and bowel transplantation are therapeutic options. A number of patients die at a young age as a consequence of septicemia [2,3,4]. MVID is autosomal recessively inherited with locus heterogeneity, where the majority of patients harbors biallelic mutations in the myosin 5b gene (MYO5B), and defects in syntaxin 3 (STX3) and syntaxin binding protein 2 (STXBP2) account for additional cases of MVID [1,5,6,7,8]. 

Up to 54% of MVID patients develop persistent cholestatic liver disease, which appears unrelated to treatment with TPN and is clinically indistinguishable from progressive familial intrahepatic cholestasis (PFIC) types I, II, IV, and V (PFIC-1, PFIC-2, PFIC-4, PFIC-5) [9,10,11]; there is persistent cholestasis with normal serum gamma-glutamyl transferase (GGT) concentrations, reduced concentrations of primary bile acids in bile, and progressive liver damage that frequently requires liver transplantation in childhood [12,13,14]. PFIC-1, PFIC-2, PFIC-4, and PFIC-5 are caused by biallelic mutations in genes encoding the canalicular membrane transporter proteins ATP8B1, the aminophospholipid flippase FIC-1 and ABCB11, the bile salt export pump (BSEP), the tight junction protein-2 (TJP2), and the transcription factor NR1H4. Recently, bi-allelic MYO5B mutations have also been identified in a small number of patients with a low-GGT PFIC phenotype in the absence of congenital diarrhea [9,10,11]; MYO5B is therefore considered both the main disease gene for MVID and to represent one of the increasing number of disease genes for low-GGT type PFIC, and the delineation PFIC6 has been suggested for this disorder [15].

Biallelic MYO5B mutations are thus identified in patients with a spectrum of clinical manifestations, ranging from intestinal disease (MYO5B-MVID) to intestinal disease combined with cholestatic liver disease in the same patients (MYO5B-MIXED), where MVID and the liver disease can be of equal or unequal medical concern, to predominant cholestatic liver disease, clinically indistinguishable from low-GGT PFIC (MYO5B-PFIC). Very recently, molecular mechanisms were proposed that can explain the occurrences of predominant intestinal, predominant hepatic and mixed phenotypes [15]. We compiled the clinical outcomes of 114 novel and previously reported patients with mostly biallelic MYO5B mutations and probed the dataset for genotype–phenotype correlation with respect to proposed disease mechanisms. Our study includes 44 novel patients with biallelic MYO5B mutations, of which 35 MYO5B mutations are novel.

Novel data will be entered into the international online registry for patients with MYO5B mutations and their phenotype information (http://www.MVID-central.org) [16]; this patient registry already contains incomplete phenotype or genotype information on a small number of patients reported in our study; our study enables straightforward complementation of these entries. 

## 2. Materials and Methods

The Department of Pediatrics I at the Medical University of Innsbruck participates in a pediatric liver transplantation program in Austria, and there is both a diagnostic and research focus on pediatric liver diseases as well as a research focus on the delineation of congenital and inherited diarrheas. Patients and patient samples are referred continuously for genetic testing to determine the etiology of the apparent hepatic or intestinal disorder. Written informed consent for molecular research investigations was obtained from the patients or from minor patients’ parents, and the studies were approved by the local ethics committee (vote No. AN2016-0029 359/4.5). 

In this study, we retrospectively identified all cases referred to our institution between 2007 and 2020 with chronic diarrhea, with cholestatic liver disease, and with both chronic diarrhea and chronic liver disease; all cases involved patients in whom we identified biallelic pathogenic or likely pathogenic MYO5B variants. We then re-contacted the referring physicians to provide a standardized, questionnaire-based follow-up on their referrals. The following data were abstracted from the medical records of each patient: family history, sex, ethnicity, parental consanguinity, age at onset of diarrhea, failure to thrive, dependence on parenteral nutrition, single- or multi-organ transplantation, characteristic histological findings of duodenal biopsies, outcome at last patient contact. Routine hepatic function criteria were determined in patients with chronic diarrhea and with chronic liver involvement. Genomic DNA was isolated from patients’ peripheral blood leukocytes by standard procedures. DNA samples from patients’ healthy parents were obtained and tested for MYO5B variant segregation with disease. 

Targeted Sanger sequencing of the MYO5B gene was conducted in 42 patients as reported previously [17,18]; MYO5B gene analysis was performed in whole-exome-sequencing (WES) data in the remaining 27 patients; WES was conducted and data processed as reported [19]; variant calling was restricted to the MYO5B region (chr18:47349156-47721542 in the hg19 reference sequence) in patients with a clinical diagnosis of MVID. In five instances, MYO5B mutation analysis had been conducted initially in a collaborating diagnostic laboratory. In patients referred with a clinically indeterminate congenital diarrhea, the genomic regions of genes known to cause forms of congenital diarrhea and immune deficiency syndromes were evaluated (gene panel list available from the authors on request). Patients with cholestatic liver disease, in whom mutations in PFIC1-3 genes had been excluded, were evaluated for MYO5B variants. Categorization and interpretation of sequence variants were based on variant allele frequencies <0.005 in public domain population databases, and predicted effects on protein function by in-silico evaluation using Polyphen2 and CADD programs [20]. The nomenclature of identified variants and patients’ genotype follows the Human Genome Variation Society guidelines (HGVS v 2.0, www.hgvs.org/mutnomen). The MYO5B variant designation is based on the NCBI reference sequence for transcript NM_001080467.3 and the genomic reference sequence NG_012925.2. The exon numbering is based on NG_033082.1. Nucleotide numbering uses +1 as the A of the ATG translation initiation codon in the reference sequence, with the initiation codon as codon 1.

**Figure 1:**
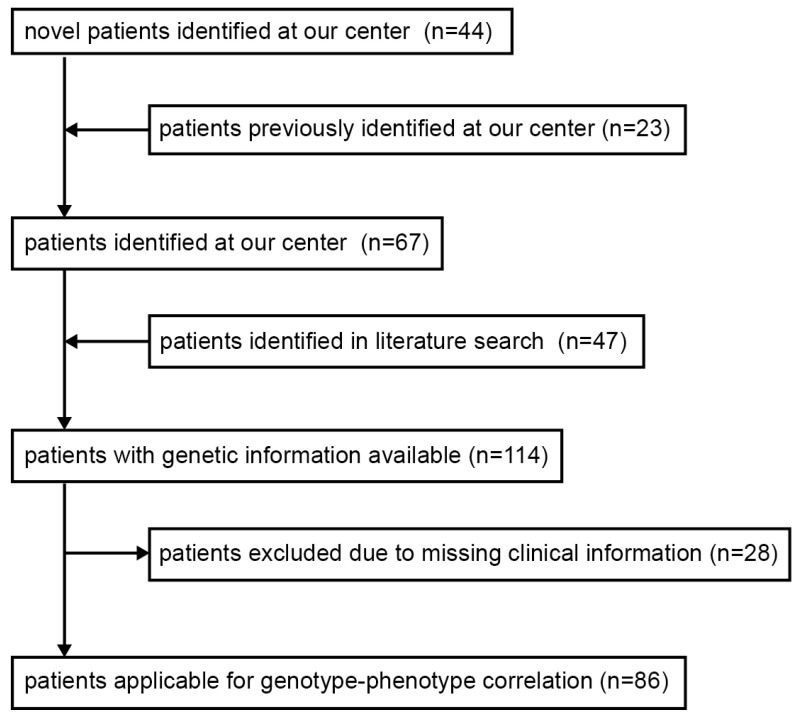
Flowchart scheme illustrating study cohort composition and selection.

We evaluated the MYO5B mutation-related data from our center together with published mutation data that were retrieved by a PubMed search with search terms “MYO5B mutations, microvillous atrophy, microvillus atrophy, microvillus inclusion disease” and in which associated phenotype data were reported. Compiled data (Figure 1) were analyzed with respect to genotype–phenotype correlations and previously proposed cellular disease mechanisms [11,15,21]. 

The statistical significance of genotype–phenotype correlations was determined using Fisher’s exact test with *p* < 0.05. 

## 3. Results

### 3.1. Study Population

In this study, we present a cohort of 114 patients with MYO5B mutations. Since the first observation of MYO5B gene mutations in 9 MVID patients [17], we have identified pathogenic and likely pathogenic MYO5B variants in a total of 67 patients at our center within the last decade. We previously reported findings in 23 of these patients [1,17,18,22]. The remaining 44 patients’ corresponding genetic and clinical data are presented in this study for the first time. From our literature search, we extracted genotype and phenotypic data of 47 additional patients with mostly biallelic MYO5B mutations [9,10,11,23,24,25,26,27,28,29,30,31,32,33] (Figure 1). 

Patients were of diverse ethnicity, and parental consanguinity was known in one third of all cases. The median age at last patient contact was 5 years (IQ-range 9 years). For the 108 patients of the cohort whose gender was known, there was no bias (62 males and 46 females, *p* = 0.24, chi-squared test).

### 3.2. Clinical Characteristics

Final diagnoses of 114 patients are shown in Table 1. Age at disease onset was known in 107 out of 114 patients; disease manifested in the first week of life in 63.6% of patients and after one year of age in 6 patients. Thirty-three out of 114 patients had died at a median age of 1 year (IQ-range 2.5 years). For 19 of 114 patients, outcome information was missing. Cause of death was mainly septicemia. Necrotizing enterocolitis, liver failure, macrophage activation syndrome, or drug resistant epilepsy with respiratory distress accounted for deaths in single cases. Twenty-two deceased patients had been diagnosed with MYO5B-MVID, and 11 patients with MYO5B-MIXED. 

25 out of 114 patients (21.9%) received single or multi-organ transplants; information regarding transplantation was not available for 8 of 114 patients. Median age at 1st transplant surgery was 3.5 years (IQ-range 1 year), median age at 2nd transplant surgery was 9.25 years (IQ-range 6.88 years); first transplants were 17 x small bowel, 2 x small and large bowel, 5 x small bowel and liver, 1 x no data. Non-transplanted MVID patients were dependent on TPN, with the exception of 4 patients who required partial PN, and four patients could be weaned completely from PN at ages of 3–7 years but developed liver disease during childhood. We obtained no information on the current treatment of 8 patients. 

Forty-one out of 114 patients had additional medical concerns, of which polyhydramnios, premature birth, failure to thrive, and renal Fanconi syndrome were seen in more than one patient. Congenital lung hypoplasia, necrotizing enterocolitis, bowel dissection, hematuria, proteinuria, calciuria, exocrine pancreatic insufficiency, low IgG level, hypothyroidism, gastroesophageal reflux disease, eczema, juvenile idiopathic arthritis, language development delay, pyramidal syndrome, epilepsy, short stature, microcephaly, and polydactyly were present in single cases. 

Detailed information on all 114 individual patients is provided in Supplementary Table S1.

### 3.3. Molecular Findings in the Study Cohort

**Figure 2:**
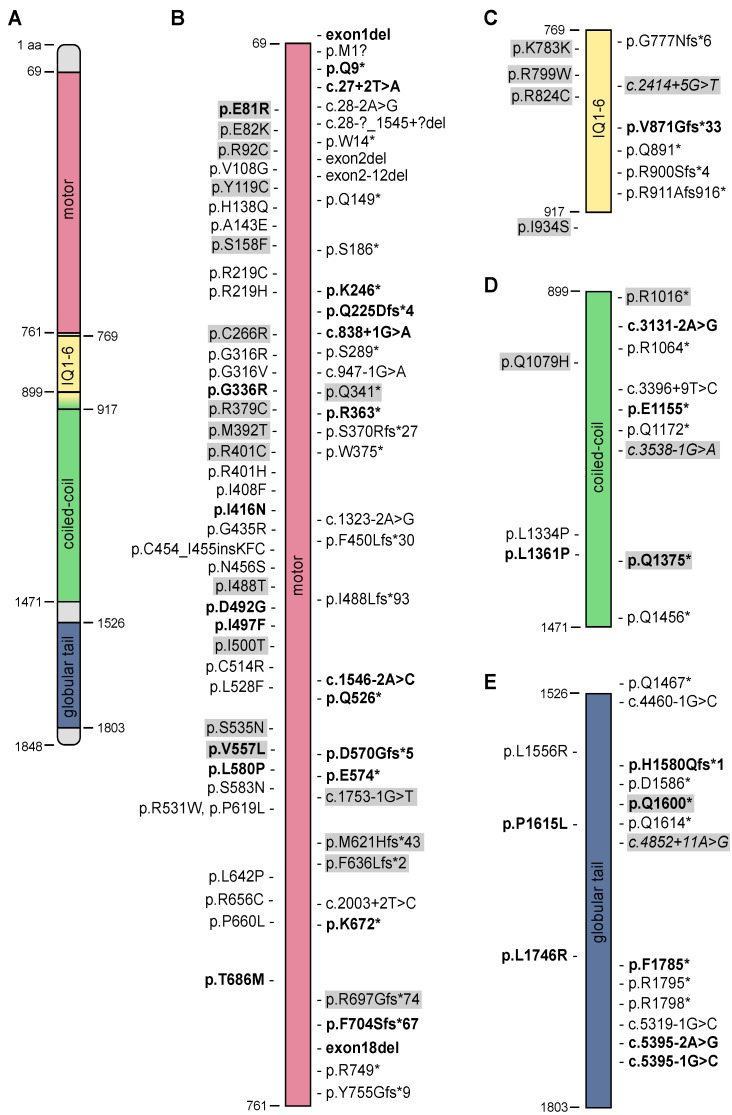
Schematic overview of myosin Vb (MYO5B) protein and annotated mutations. (**A**) Full-length MYO5B with domain-positions in aminoacids. Protein length and domain annotation are shown as in UniProt (http://www.uniprot.org/uniprot/Q9ULV0). The Motor (**B**), IQ 1–6 (**C**), coiled-coil (**D**) and globular tail domain (**E**) are shown separately. (**B**–**E**) Missense mutations are annotated on the left of the respective domain and truncating mutations on the right. Mutations on opaque background are associated with liver disease and novel mutations first described in this study are in bold letters. Mutations in italic letters are in-silico predicted to result in truncated or abnormal protein. * denotes a premature stop codon.

The 114 reported patients implicate 114 distinct MYO5B alleles in causing disease (Figure 2), of which we report 35 novel mutations here. These mutations include 4 intragenic deletions of one or several exons, 16 noncoding/splicing mutations, and 45 frameshifts/nonsense/initiation codon mutations, which are all predicted to lead to MYO5B truncation, as well as 49 missense mutations and one in-frame insertion. 

An analysis of 67 previously reported patients along with the 44 newly identified patients with MYO5B mutations is consistent with the reported autosomal recessive inheritance of disease (Supporting Information Table S2). Forty-seven of 114 patients (41%) were homozygous for MYO5B mutations, and all but 7 of the remainder were compound-heterozygotes. Seven patients displayed characteristic phenotypes, but only one mutated MYO5B allele was found with the current techniques that address only exonic and flanking intronic gene sequences. The large proportion of homozygous individuals is consistent with the high incidence of MYO5B patients amongst consanguineous marriages.

Missense mutations cluster in the head domain of MYO5B (Table 2), while truncating mutations are more uniformly distributed over the whole MYO5B protein. Missense mutations are found in all subdomains of the myosin head domain. With the exception of the arginine residue at position 401, which is mutated to cysteine in MYO5B-PFIC, and is mutated to histidine in MYO5B-MVID, the MYO5B-PFIC-associated and MYO5B-MIXED-associated missense mutations affect distinct amino acid residues (Figure 2). 

### 3.4. Genotype–Phenotype Correlation and Mutations of Particular Interest

Out of the cohort of all 114 patients, 28 were excluded from our genotype–phenotype correlation due to incomplete phenotyping (Figure 1). The remaining 86 patients were allocated to 3 different groups according to their phenotypes: MYO5B-PFIC, MYO5B-MVID, and MYO5B-MIXED. For 78 out of these 86 cases, the biallelic mutational genotypes were known (Table 3). A detailed list of all patients’ genotypes is provided in Table 4. 

The MYO5B mutational and genotypic spectrum in patients with the predominant hepatic phenotype, i.e., MYO5B-PFIC, is different from the spectrum in patients with the predominant intestinal phenotype, MYO5B-MVID (Table 4). Biallelic truncating genotypes are significantly more frequent in the MYO5B-MVID group than in the MYO5B-PFIC group (Fisher’s exact test, *p* < 0.001).

It is noteworthy that each MYO5B genotype was only observed within one phenotype category, implying a strong genotype–phenotype correlation. In addition, only four out of 114 different MYO5B mutations were observed in different phenotype categories, including p.R1016 * and p.M392T. Interestingly, arginine-401 was mutated to cysteine (p.R401C) in one patient with cholestatic liver disease [11], while being mutated to histidine (p.R401H) in two patients with MVID [18]. 

MYO5B-PFIC was reported in 27 patients, of whom 75% developed the disorder within the first year of life. Twenty-eight patients were diagnosed with MYO5B-MIXED. However, exact differentiation between TPN-associated liver disease and MYO5B-PFIC cannot be clearly made in every case.

Few mutations were seen in more than two families. The p.P660L mutation represents a known founder mutation among Navajo Indian patients with MVID and cholestasis, i.e., MYO5B-MIXED [25], and was counted only once in this study. The p.R1016 * mutation was the most common mutation in our study and was present in 10 disease alleles; it was identified in patients of Han Chinese, Anglo-American, and Hispanic ancestry. The corresponding DNA point mutation affects a CpG island, which might be prone to independent mutational events. This mutation was present in homozygous state in a single patient and compound-heterozygous with an exon 2 deletion in another patient, both times associated with MYO5B-MVID. Taking p.R1016 * mutation as an example of the large number of early premature stop codons observed in MYO5B, this mutation is expected to trigger nonsense-mediated mRNA decay and to abolish protein production. 

It is of interest that the p.R1016 * mutation was seen in compound-heterozygous state with a p.M392T mutation in two unrelated patients with a notable phenotype: clinical and histopathological characteristics of MVID were documented in the first years of life in both patients, but MVID resolved completely during childhood, whereas MYO5B-PFIC manifested subsequently at ages of 7 and 15 years in these patients. The former patient received a liver transplant for cholestatic liver disease at age 11 years, the latter received an endogenous internal diversion. Both compound-heterozygotes were on a normal diet at last contact at 11 and 24 years of age, respectively; the older patient has been reported twice [26,32], and the genetic testing was performed in our laboratory. This transient-MVID and late-MYO5B-PFIC phenotype was encountered in two other patients with distinguishable genotypes, p.E82K/p.E82K and p.H81/p.Q1600 *. Regarding genotype p.E82K/p. E82K, two siblings of 9 and 5 years were also diagnosed with MVID postnatally, and MVID resolved completely; so far, no hepatic disease occurred.

Mutation p.R656C, predicted to be functionally damaging by all in-silico tools, is a candidate founder mutation in Turkish and Arab patients. In 3 patients from the United Arab Emirates, the MYO5B p.R656C allele harbored an additional p.P1615L mutation on the same allele. Three homozygotes for p. R656C presented with MYO5B-MIXED and were alive at ages 8, 12, and 17 years. The youngest p.R656C homozygote died at age 3.5 years, and chronic cholestatic liver involvement was not recorded but cannot be excluded. Two compound-heterozygotes for p.R656C and an early truncating mutation, p.K672*, had presented with MYO5B-MVID until their deaths at 7 and 9 months of age.

## 4. Discussion

Biallelic MYO5B mutations have emerged as the major cause of MVID since 2008 [17,18,23,24,25]. Currently, MVID represents one disease entity among approximately 50 monogenic disorders that are termed congenital diarrhea and enteropathies (CODEs). CODEs are typically associated with persistent and severe diarrhea presenting in the first weeks of life and with feeding intolerance and malabsorption [34]. CODEs require significant dietary and therapeutic interventions, including specialized formulas or PN to sustain appropriate growth, electrolyte, and nutrient balance. MVID was recognized as an entity on clinical and histopathological grounds in 1978 [2]. Marked but variably severe hepatic involvement was recognized in a significant percentage of MVID patients thereafter, which is considered at least in part to represent a consequence of TPN and of a disrupted enterohepatic circulation in young children [4,35]. Hepatic involvement most often manifested in the first year of life and was similar to low-GGT PFIC. Recently, four studies reported a small series of patients with PFIC who were negative for mutations in the known PFIC genes and harbored biallelic MYO5B mutations, or pathogenic monoallelic MYO5B mutations, where a mutation on the second allele had not been detected with the current diagnostic techniques [9,10,11,26]. 

Since our first reporting of MYO5B mutations in MVID, we identified MYO5B mutations in 67 patients. In this study, we compile genetic and clinical findings from 44 new patients with MYO5B mutations and from 47 patients with clinical and genetical findings from the literature. Our review of a total of 114 patients demonstrates that there are indeed 3 distinctive phenotypes associated with MYO5B genotypes: MYO5B-PFIC, pure MYO5B-MVID, and MYO5B-MIXED. Our study shows that there is a nearly complete genotype–phenotype correlation, i.e., each MYO5B genotype belongs to one of the above classes, as was suggested in previous studies with fewer patients with known genotypes and in functional studies [7,11,15]. 

How do different biallelic MYO5B genotypes cause isolated hepatic or intestinal disease, or concomitant hepatic and intestinal disease? An evaluation of our comprehensive cohort of patients with known MYO5B genotypes and their phenotypes is consistent with the following hypotheses [11,15,21]:

(1) Biallelic mutations that predict nonsense-mediated mRNA decay are always associated with MYO5B-MVID. This indicates that the MVID phenotype is caused by a loss-of-function of MYO5B, which causes clinical symptoms via disrupted enterocyte polarization [1,17,22,36]. The small number of missense mutations associated with MYO5B-MVID might all lead to misfolding and MYO5B degradation, also resulting in lack of MYO5B protein. The loss of MYO5B motor function alone does not cause liver disease.
(2) Contrastingly, a distinct set of missense mutations is identified in patients with MYO5B-PFIC, and these mutant MYO5B proteins are expressed, as has been shown for two of these missense mutations, p.C266R, and p.S158F [11,15,37]. Apparently, intestinal MYO5B function of these mutant proteins is preserved, at least to the degree in that such biallelic missense mutations allow sufficient degree of enterocytic function to maintain intestinal autonomy. However, displacement of bile canalicular transporters to the cytoplasm of hepatocytes was shown in liver biopsies of patients with MVID presenting with cholestasis and homozygous missense mutations p. P660L [37] as well as in patients with MYO5B-PFIC with the homozygous missense mutations p. C266R [11] and p.S158F mutation [15]. Mutagenesis experiments showed that the disrupting effect of PFIC-associated MYO5B motor domain mutants on the localization of canalicular proteins was critically dependent on their preserved ability to interact with active RAB11a, as loss of MYO5B did not affect RAB11-dependent vesicle trafficking [15].
(3) The conclusion from recent in vitro and in vivo studies is indirectly supported by our genotype–phenotype correlation, which shows that MYO5B is not required for the correct localization of hepatic canalicular proteins. It was shown that PFIC-associated MYO5B mutants require active RAB11a for their disruptive effect on canalicular protein localization, and this previous study indicated a direct and simple explanation for the observed genotype–phenotype correlation in patients with MYO5B mutations.
(4) Patients with MYO5B-MIXED most often present with a missense or late-truncating mutation in trans with a loss-of-function mutation in MYO5B. In these instances, the presence of a single mutant expressed protein cannot prevent MVID development in enterocytes and causes cholestatic liver disease by putatively interfering with RAB11- or RAB8-dependent [38] processes.

**Figure 3:**
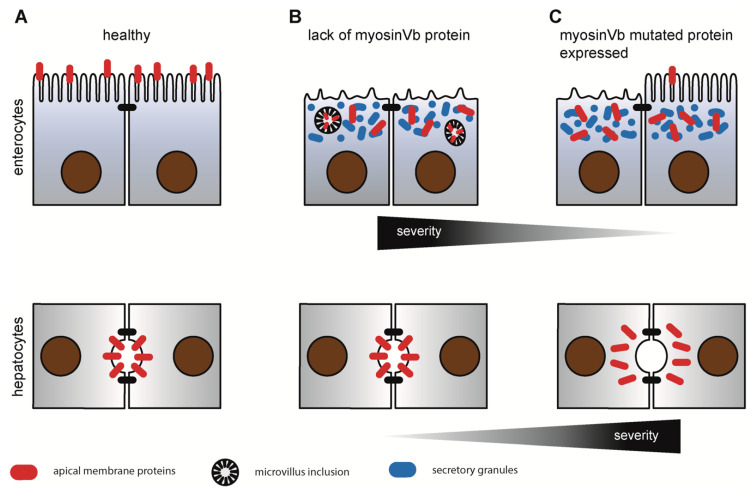
Schematic overview of enterocytic and hepatocytic phenotypes observed upon mutations in myosin Vb (MYO5B). (**A**) healthy enterocytes and hepatocytes establish proper epithelial differentiation. Apical proteins (e.g., transmembrane transporters and enzymes; red) localize at the apical brush border in enterocytes or bile canalicular membrane in hepatocytes. (**B**) lack of MYO5B results in MYO5B-MVID: disrupted apical brush border, subapical “secretory” granules (blue), mislocalization of apical proteins (red) and microvillus inclusions. Hepatocyte function is unaffected. (**C**) Expressed mutated MYO5B results in varying degree of disease-severity and enterocytic phenotypes but leads to mislocalisation of apical bile transporters and cholestasis in hepatocytes.

The correlation between loss-of-function and expressed mutant proteins is displayed schematically in Figure 3.

Very rarely do patients present with transient MVID in the first years of life and develop liver disease in childhood. Interestingly, this appears to occur only with certain MYO5B genotypes. It might be speculated that MVID is present at times where nutritional load per surface area and intestinal growth are highest in the first years of life. Liver disease might ensue with increasing load of the enterohepatic circulation. Along similar lines, the intestinal microbiome is a relevant modulator of bile acids and the enterohepatic circulation, via bile acid modification, and only fully matures during the first years of life [39]. This then might lead to the misbalance of a fragile hepatocytic homeostasis and result in MYO5B-PFIC. It is known that therapeutic intervention can change the MYO5B-related phenotype. For example, MYO5B-MVID patients develop MYO5B-PFIC following a bowel transplantation, supposedly due to an altered enterohepatic circulation [7]. 

The majority of MYO5B mutations were observed in individual patients or families, highlighting the need for a complete MYO5B sequence analysis on suspicion of MYO5B-MVID, MYO5B-PFIC, and MYO5B-MIXED. Such an analysis needs to incorporate testing for copy-number-variants (CNVs) as intragenic exon deletions constitute 4 of 114 distinct disease variants and represent 5 out of 230 disease alleles. CNV calling can be included in any next-generation-sequencing pipeline suited to analyzing gene panels for congenital diarrheas and for pediatric cholestatic disorders. 

## 5. Conclusions

Our data support the concept that (1) MVID results from the loss of MYO5B function in enterocytes, (2) that a complete lack of protein and greatly truncated MYO5B proteins do not cause primary liver disease, (3) that solitary primary cholestatic liver disease results from the expression of mutant MYO5B proteins that cause aberrant protein–protein interactions in hepatocytes, (4) and that expressed non-functional MYO5B protein causes both intestinal and hepatic disease.

The existence of an experimental mouse model for MYO5B disease will provide a useful platform for testing mechanistic hypotheses [22,40,41].

## Figures and Tables

**Table 1:** Baseline phenotype characteristics and outcome of 107 patients with biallelic and 7 patients with mono-allelic Myosin Vb (MYO5B) variants.

**MYO5B-PFIC**	23	20.2%
**MYO5B-MIXED**	32	28.1%
**MYO5B-MVID**	31	27.2%
**Missing information**	28	24.5%
**total**	114	100%

**Table 2:** MYO5B missense mutation per domain distribution.

		Domain				
		Head	IQ	Coiled Coil	Tail	Total
Mutation	count (%)	37 (80.4)	4 (8.7)	2 (4.4)	3 (6.5)	46 (100)
Amino acid residues	count (%)	692 (41.9)	130 (7.9)	554 (33.5)	277 (16.7)	1653 (100)

**Table 3:** Biallelic MYO5B genotypes (n = 78) observed within 3 phenotype categories.

		Genotype				Total
			Biallelic Missense	Biallelic Truncating	Missense-Truncating	
**Phenotype**	MYO5B-MVID	count (%)	9 (31.0)	15 (51.7)	5 (17.3)	29 (100)
	MYO5B-PFIC	count (%)	11 (57.9)	0 (0)	8 (42.1)	19 (100)
	MYO5B-MIXED	count (%)	12 (40.0)	11 (36.7)	7 (23.3)	30 (100)
total			32	26	20	78

**Table 4:** MYO5B genotypes (*n* = 86) associated with MYO5B-PFIC, MYO5B-MVID, MYO5B-MIXED.

MYO5B-PFIC					MYO5B-MVID					MYO5B-MIXED				
Study	ID	cDNA	Protein	Class	Study	ID	cDNA	Protein	Class	Study	ID	cDNA	Protein	Class
this study	10D2367	c.1669G > T	p.V557L. p.V557L	Mis Mis	this study	18D1383	c.2014A > T c.2014A > T	p.K672 * p.K672 *	Tru Tru	this study	18D4596	c.1247T > A c.1247T > A	p.I416N p.I416N	Mis Mis
this study	11D1388	c.1669G > T	p.V557L. p.V557L	Mis Mis	this study	10D0098	c.3190C > T c.3514C > T	p.R1064 * p.Q1172 *	Tru Tru	this study	10D0028	c.1475A > G c.1475A > G	p.D492G p.D492G	Mis Mis
[26]	pat 1	c.274C > T 1463T > C	p.R92C p.I488T	Mis Mis	this study	16D2984	c.4399C > T c.4399C > T	p.Q1467 * p.Q1467 *	Tru Tru	this study	11D2081	c.[1966C > T;4844C > T] c.[1966C > T;4844C > T]	p.[R656C;P1615L] p.[R656C;P1615L]	Mis Mis
[26]	pat 3	c.2470C > T	p.R824C p.R824C	Mis Mis	this study	15D1631	c.1323-2A > G c.1323-2A > G	splicing splicing	Tru Tru	this study	12D1383	c.[1966C > T;4844C > T] c.[1966C > T;4844C > T]	p.[R656C;P1615L] p.[R656C;P1615L]	Mis Mis
[11]	pat 4	c.3237G > C c.1604G > A	p.Q1079H p.S535N	Mis Mis	this study	09D0802	c.3046C > T c.3046C > T	p.R1016 * p.R1016 *	Tru Tru	this study	17D1468	c.3131-2A > G c.3131-2A > G	splicing splicing	Mis Mis
[11]	pat 5	c.796T > C	p.C266R p.C266R	Mis Mis	this study	10D0875	c.736C > A c.2612del	p.K246 * p.V871Gfs * 33	Tru Tru	[17]	14483	c.1966C > T c.1966C > T	p.R656C p.R656C	Mis Mis
[11]	pat 6	c.1748G > A c.2801T > G	p.S583N p.I934S	Mis Mis	this study	11D0567	c.2014A > T c.2014A > T	p.K672 * p.K672 *	Tru Tru	[18]	106	c.502G > A c.502G > A	p.G168R p.G168R	Mis Mis
[11]	pat 10	c.2470C > T	p.R824C p.R824C	Mis Mis	this study	11D1811	c.838 + 1G > A c.4740_4741del	splicing p.H1580Qfs * 1	Tru Tru	[18]	S285C1	c.4667_4668TT > GC c.4667_4668TT > GC	p.L1556R p.L1556R	Mis Mis
[10]	pat 1	c.274C > T c.2395C > T	p.R92C p.R799W	Mis Mis	this study	12D2180	c.2T > A c27 + 2T > A	p.M1? splicing	Tru Tru	[18]	107	c.1303G > A c.1303G > A	p.G435R p.G435R	Mis Mis
[10]	pat 2	c.1499T > C c.1925T > C	p.I500T p.L642P	Mis Mis	this study	17D2697	c.25C > T exon 1del	p.Q9 * start removal	Tru Tru	[28]	pat 2	c.1222A > T c.1582C > T	p.I408F p.L528F	Mis Mis
[10]	pat 3	c.356A > G	p.Y119C p.Y119C	Mis Mis	this study	18D0872	c.2245C > T exon 1del	p.R749 * start removal	Tru Tru	[28]	pat 3	c.1222A > T c.1582C > T	p.I408F p.L528F	Mis Mis
this study	14D0856	c.274C > T c.4123C > T	p.R92C p.Q1375*	Mis Tru	this study	GOSH 1	c.1087C > T c.1087C > T	p.R363 * p.R363*	Tru Tru	this study	13D2752	c.445C > T c.5383C > T	p.Q149* p.R1795*	Tru Tru
this study	14D0857	c.274C > T c.4123C > T	p.R92C p.Q1375 *	Mis Tru	this study	GOSH 2	c.3046C > T c.5354_5355del	p.R1016 * p.F1785*	Tru Tru	this study	10D1077	c.1323-2A > G c.1323-2A > G	splicing splicing	Tru Tru
[11]	pat 8	c.437C > T c.3046C > T	p.S158F p.R1016 *	Mis Tru	[18]	114	c.1110_1113del c.4755dup	p.S370Rfs * 27 p.D1586*	Tru Tru	this study	11D1983	c.5395-1C > G c.5395-1C > G	splicing splicing	Tru Tru
[11]	pat 9	c.437C > T c.3046C > T	p.S158F p.R1016 *	Mis Tru	[18]	115	c.2003 + 2A > G c.2003 + 2A > G	splicing splicing	Tru Tru	this study	EN1	c.5395-1C > G c.5395-1C > G	splicing splicing	Tru Tru
[26]	pat 2	c.274C > T c.1860dupT	p.R92C p.M621Hfs * 43	Mis Tru	this study	14D0669	c.1966C > T c.2014A > T	p.R656C p.K672*	Mis Tru	this study	GOSH 3	c.1576C > T c.2111del	p.Q526 * p.F704Sfs*67	Tru Tru
[10]	pat 4	c.2470C > T c.1135C > T;c.1906-2A > G	p.R824C p.R379C;F636Lfs * 2	Mis Tru	this study	18D4787	c.1966C > T c.2014A > T	p.R656C p.K672 *	Mis Tru	this study	GOSH 4	c.672_673del c.672_673del	p.Q225Dfs * 4 p.Q225Dfs * 4	Tru Tru
[10]	pat 5	c.2395C > T c.1753-1G > T	p.R799W splicing	Mis Tru	this study	10D1884	c.1591C > T;c.1856C > T c.5395-2A > G	p.R531W; p.P619L splicing	Mis Tru	[17]	14484	c.5392C > T c.5392C > T	p.R1798 * p.R1798 *	Tru Tru
[11]	pat 3	c.1201C > T c.1021C > T	p.R401C p.Q341*	Mis Tru						[18]	112	c.866C > A c.4840C > T	p.S289 * p.Q1614*	Tru Tru
					this study	EN6	c.947G > T c.4082T > C	p.G316V p.L1361P	Mis Mis	[18]	121	c.947-1G > A c.947-1G > A	splicing splicing	Tru Tru
[11]	pat 1	c.3538-1G > A§ c.2414 + 5G > T&	splicing splicing	In-frame del In-frame del	[8]	14D2264	c.414C > A c.414C > A	p.H138Q p.H138Q	Mis Mis	[33]	pat 1	c.1462del c.1462del	p.I488Lfs * 93 p.I488Lfs * 93	Tru Tru
[11]	pat 2	c.3538-1G > A§ c.2414 + 5G > T&	splicing splicing	In-frame del In-frame del	[27]	C25	c.505 A > G c.505 A > G	p.K169E p.K169E	Mis Mis	[22]	19D0612	c.1323-2A > G c.1323-2A > G	splicing splicing	Tru Tru
[26]	pat 4	c.1175T > C c.2349A > G	p.M392T p.K783 =	Mis ?	[28]	pat 6	c. 656G > A c.4028T > C	p.R219H p.L1343P	Mis Mis	this study	11D0303	c.1489A > T c.1720G > T	p.I497F p.E574 *	Mis Tru
										this study	13D2757	c.1739T > C c.1110_1113del	p.L580P p.S370Rfs * 27	Mis Tru
					[28]	pat 7	c.1347delC c.3163-3165dup	p.F450Lfs * 30 ?	Mis ?	[18]	108	c.428C > A c.42G > A	p.A143E p.W14 *	Mis Tru
[11]	pat 7	c.2090del c.4852 + 11A > G$	p.R697Gfs * 74 splicing	Mis ?	[28]	pat 8	c.1347delC c.3163-3165dup	p.F450Lfs * 30 ?	Mis ?	[26]	pat 6*	c.244G > A	p.E82K p.E82K	Mis Mis
					[8]	pat 1* 15D3187	c.244G > A	p.E82K p.E82K	Mis Mis	this study	EN9	c.242A > G c.4798C > T	p.H81R p.Q1600 *	Mis Tru
					This study	13D1128 *	c.244G > A	p.E82K p.E82K	Mis Mis	this study	EN10	c.1175T > C c.3046C > T	p.M392T p.R1016 *	Mis Tru
					This study	09D1219	c.1087C > T exon 18del	p.R363 * ?	Tru tru	[26]	pat 5	c.1175T > C c.3046C > T	p.M392T p.R1016 *	Mis Tru
					This study	12D0574	c.[1966C > T; 4844C > T] c.[1966C > T; 4844C > T]	p.[R656C; P1615L] p.[R656C; P1615L]	Mis Mis					
					This study	14D0124	c.2057C > T c.2057C > T	p.T686M p.T686M	Mis Mis	[30]	pat 1	c.2729_2731delC ?	p.R911Afs916 * ?	Tru ?
					This study	13D2411	c.2057C > T c.2057C > T	p.T686M p.T686M	Mis Mis	[28]	pat 1	c.2259-2262dup ?	p.Y755Gfs * 9 ?	Tru ?
					This study	EN5	c.1175T > C c.3046C > T	p.M392T p.R1016 *	Mis Tru	This study	13D2706	c.1708dup c.1006G > A	p.D570Gfs * 5 p.G336R	Tru Mis

§ Deletes the intron-26 acceptor splice-site; predicts exclusion of exon 27 and a protein with a 22-amino-acid inframe deletion. & Weakens the motif of the exon 19-intron 19 donor splice-site; usage of an upstream splice-site is predicted, with the consequence of exclusion of the terminal 27 bp of exon 19 and a 9-amino-acid inframe deletion of the protein. $ No in-silico splicing effect predicted; phase of the genotype was not determined; both identified variants might reside on the same allele with an unknown variant on the other allele that might allow for protein expression. Red font: Mutations that appear in more than one group of phenotypes. Mis Mis, biallelic missense mutations; Mis Tru, compound-heterozygous for missense and truncating mutation, Tru Tru, biallelic truncating mutations; ?, mutation on one allele not identified or consequence ambiguous.

## Data Availability

The data presented in this study are available on reasonable request from the corresponding author.

## Supplementary Materials

The following are available online at https://www.mdpi.com/2077-0383/10/3/481/s1, Table S1: Detailed information on all 114 individual patients.
